# Transferable Resistance to Highest Priority Critically Important Antibiotics for Human Health in *Escherichia coli* Strains Obtained From Livestock Feces in Uruguay

**DOI:** 10.3389/fvets.2020.588919

**Published:** 2020-11-19

**Authors:** Nadia Coppola, Bibiana Freire, Ana Umpiérrez, Nicolás F. Cordeiro, Pablo Ávila, Gustavo Trenchi, Gustavo Castro, María Laura Casaux, Martín Fraga, Pablo Zunino, Inés Bado, Rafael Vignoli

**Affiliations:** ^1^Departamento de Bacteriología y Virología, Facultad de Medicina, Universidad de la República, Montevideo, Uruguay; ^2^Unidad Académica Animales de Granja, Facultad de Veterinaria, Universidad de la República, Montevideo, Uruguay; ^3^Departamento de Microbiología, Instituto de Investigaciones Biológicas Clemente Estable, Montevideo, Uruguay; ^4^Veterinario de Libre Ejercicio, Montevideo, Uruguay; ^5^Plataforma de Investigación en Salud Animal, Instituto Nacional de Investigación Agropecuaria, Colonia, Uruguay

**Keywords:** swine, poultry, ESBL, *E. coli*, MCR-1, CTX-M-8, CMY-2, *qnrB19*

## Abstract

The aim of this work was to detect *Escherichia coli* isolates displaying resistance to oxyimino-cephalosporins, quinolones, and colistin in feces from livestock in Uruguay. During 2016–2019, fecal samples from 132 broiler and layer chicken flocks, 100 calves, and 50 pigs, were studied in Uruguay. Samples were cultured on MacConkey Agar plates supplemented with ciprofloxacin, ceftriaxone, or colistin. *E. coli* isolates were identified by mass spectrometry and antibiotic susceptibility testing was performed by disk diffusion agar method and colistin agar test. Antibiotic resistance genes were detected by polymerase chain reaction and sequencing. The most frequently detected resistance gene was *qnrB19*, recovered from 87 animals. Regarding plasmid-mediated quinolone resistance genes, *qnrS1* was the second in prevalence (23 animals) followed by *qnrE1*, found in 6 chickens and two calves. Regarding resistance to oxyimino-cephalosporins, 8 different β-lactamase genes were detected: *bla*_CTX−M−8_ and *bla*_CMY−2_ were found in 23 and 19 animals, respectively; next, *bla*_CTX−M−2_ and *bla*_SHV−12_ in 7 animals each, followed by *bla*_CTX−M−14_ in 5, *bla*_CTX−M−15_ and *bla*_SHV2a_ in 2, and *bla*_CTX−M−55_ in a single animal. Finally, the *mcr-1* gene was detected only in 8 pigs from a single farm, and in a chicken. Isolates carrying *bla*_CMY−2_ and *bla*_SHV−12_ were also found in these animals, including two isolates featuring the *bla*_CMY−2_/*mcr-1* genotype. To the best of our knowledge, this is the first work in which the search for transferable resistance to highest priority critically important antibiotics for human health is carried out in chickens and pigs chains of production animals in Uruguay.

## Introduction

The interaction between humans and animals is quite diverse and may lead to cases of zoonosis and/or anthropozoonosis (1). Over the past decades the aforementioned interaction has constantly increased worldwide partly due to animal husbandry practices, the growth of the companion animal market, climate change, and ecosystem disruption. In this context, bacterial transmission may occur through food products (e.g., meat or eggs) or through direct contact, in particular in farmers, veterinarians, or abattoir workers (2).

As the human-animal connection escalates, so does the threat of pathogen spread (2, 3). With current rapid transport systems, a pathogen emerging today in any given country can easily be carried unnoticed in people, animals, plants, or food products, to distant parts of the world in <24 h (4).

On the other hand, anthropogenic changes to the ecosystem increase the number of shared habitats between humans and animals, thus exposing both to new pathogens. In this regard, several authors have described the occurrence in humans and several animal species of the pandemic strain, *Escherichia coli* 025:H4 ST131, carrying the extended-spectrum beta-lactamase CTX-M-15. This particular event indicates an interspecies transmission from humans to pets and livestock and has been particularly described across Europe (5).

Recently, the WHO, FAO, and OIE organizations have coined the term “One Health” which regards the environment and human and animal health as a single entity. In this context, antibiotic resistance is a major concern (6).

The highest consumption of antibiotics occurs in animal husbandry, reaching in several countries 80% of the annual total antimicrobial consumption, with a higher consumption estimated by the year 2030. In this sense, the food industry may be accountable for the spread and increase of antibiotic resistance mechanisms (6, 7).

The impact on humans of antibiotic-resistant bacteria of animal origin is reflected in the increase of enteropathogens such as *Salmonella* spp. resistant to oxyimino-cephalosporins and fluoroquinolones, responsible among others for severe pediatric infections. Moreover, resistance to azithromycin and ciprofloxacin has been noted in species such as *Campylobacter jejuni*, and ESBL-producing *Escherichia coli* (8). Particularly in the United States, enterobacteria are responsible for 140,000 healthcare-associated infections annually, with 26,000 infections attributable to ESBL-producing enterobacteria, representing 19% of hospital-acquired infections.

In this respect, foodborne infections due to these resistant enteropathogens are a risk to humans due to possible therapeutic failure (8). Evidence indicates that a reduction in antibiotic consumption in animal husbandry would lead to a decrease in bacterial antibiotic resistance levels (9).

Antibiotics, in animal husbandry, are mainly used to treat infections, furthermore, they are also used as a prophylactic, through group treatment by incorporating them in drug premixes at relatively high concentrations, and more worryingly as growth promoters (although this practice has already been banned in some countries) (8).

Particularly noteworthy is the emergence of transferable resistance to critical antibiotics of highest priority for human medicine such as oxyimino-cephalosporins, fluoroquinolones and polymyxins, due to: extended spectrum β-lactamases (ESBLs) and plasmidic cephalosporinases (pAmpC); plasmid-mediated quinolone resistance (PMQR) genes [e.g., *qnr* and *aac(6*′*)-Ib-cr*]; and *mcr* alleles, respectively (10).

Infections caused by multidrug resistant microorganisms (MRM) lead to longer hospital stays (6.4–12.7 days), increased morbimortality (6.5%) and elevated economic costs (18,588–29,069 US$ per patient) (11, 12). Additionally, the occurrence of MRM in production animals may result in economic losses in trade and agriculture commerce; an example of the latter was observed in Norway, where the presence of resistant *E. coli* in retail chicken meat resulted in a 20% decrease in sales (13).

In Uruguay, measures have been taken to restrict the use of antibiotics, both in human health and in animal husbandry. The use of antibiotics as growth promoters in the latter is forbidden, and cattle under treatment are not destined for human consumption (https://www.proa.hc.edu.uy/), (Decree N° 98/011). In addition, in March 2019 Decree 141/019 was established, prohibiting the import, export, manufacture, sale, use, possession and marketing of veterinary products containing the substance “colistin” in its composition, either alone or associated with other chemicals (either as raw material or finished product), or incorporated into animal feed.

Finally, in 2017 Umpierrez et al. reported for the first time the occurrence of antibiotic- resistance genes in *E. coli* isolated from cattle in Uruguay. In that study, the authors detected multidrug-resistant *E. coli* isolated from calves carrying *bla*_CTX−M−14_ and PMQR genes, among other resistance determinants (14).

The aim of this work was to detect *E. coli* isolates displaying resistance to oxyimino-cephalosporins, quinolones, and colistin in feces from livestock in Uruguay.

## Materials and Methods

### Sampling and Transport

During 2016–2019, fecal samples from 282 animals were studied in Uruguay: 100 from calves, 132 from broiler and layer chicken flocks and 50 from pigs of different ages; samples were obtained from five, 13, and five establishments, respectively. Between 5 and 20 animals were sampled in each establishment (see Table 1).

**Table 1:** Main results from the studied establishments.

	**Establishment**	**Studied animals**	**Animals w/cro R**	**Animals w/cip R**	**Animals w/col R**	**Selected *E.coli***	**Resistance genes**			**Genotypes detected**
							***Bla***	**PMQR**	***mcr***	
Calves	C1	20	0	8	0	11	–	*qnrB19*^(3)^	–	*qnrB19*^(3)^
	C2	20	0	5	0	6	–	*qnrB19*^(2)^	–	*qnrB19*^(2)^
	C3	20	0	16	0	20	–	*qnrB19*^(6)^	–	*qnrB19*^(6)^
	C4	20	0	7	0	7	–	–	–	–
	C5	20	1	19	0	29	${\mathrm{bla}}_{\text{CTX}-\text{M}-15}^{\left(1\right)}$	*qnrB19*^(8)^, *qnrE1*^(2)^, *qnrS1*^(1)^	–	*bla*_CTX−M−15_/*qnrB19*^(1)^, *qnrB19*^(7)^, *qnrE1*^(2)^, *qnrS1*^(1)^
	Total	100	1	55	0	73	1	22	0	
Poultry	P1	17	3	12	0	16	${\mathrm{bla}}_{\text{CTX}-\text{M}-2}^{\left(3\right)}$	*qnrB19*^(6)^, *qnrE1*^(5)^	–	${\mathrm{bla}}_{\text{CTX}-\text{M}-2}^{\left(2\right)}$, *bla*_CTX−M−2_/*qnrE1*^(1)^, *qnrE1*/*qnrB19*^(2)^, q*nrB19*^(4)^, *qnrE1*^(2)^
	P2	10	3	9	0	12	${\mathrm{bla}}_{\text{CTX}-\text{M}-2}^{\left(3\right)}$	*qnrB19*^(4)^	–	${\mathrm{bla}}_{\text{CTX}-\text{M}-2}^{\left(2\right)}$, *bla*_CTX−M−2_/*qnrB19*^(1)^, *qnrB19*^(3)^
	P3	5	2	2	0	3	${\mathrm{bla}}_{\text{CTX}-\text{M}-8}^{\left(1\right)}$, ${\mathrm{bla}}_{\text{CTX}-\text{M}-55}^{\left(1\right)}$	*qnrB19*^(2)^	–	${\mathrm{bla}}_{\text{CTX}-\text{M}-8}^{\left(1\right)}$, *qnrB19*^(1)^, *bla*_CTX−M−55_/*qnrB19*^(1)^
	P4	10	1	6	0	10	${\mathrm{bla}}_{\text{CMY}-2}^{\left(1\right)}$	*qnrB19*^(8)^	–	${\mathrm{bla}}_{\text{CMY}-2}^{\left(1\right)}$, *qnrB19*^(8)^
	P5	10	0	10	0	11	–	*qnrB19*^(8)^	–	*qnrB19*^(8)^
	P6	10	0	10	1	11	–	*qnrB19*^(1)^	*mcr-1*^(1)^	*qnrB19*/*mcr-1*^(1)^
	P7	10	0	9	0	15	–	*qnrB19*^(9)^	–	*qnrB19*^(9)^
	P8	10	6	4	0	9	${\mathrm{bla}}_{\text{CMY}-2}^{\left(5\right)}$, ${\mathrm{bla}}_{\text{CTX}-\text{M}-2}^{\left(1\right)}$	*qnrB19*^(1)^	–	${\mathrm{bla}}_{\text{CMY}-2}^{\left(5\right)}$, ${\mathrm{bla}}_{\text{CTX}-\text{M}-2}^{\left(1\right)}$, *qnrB19*^(1)^
	P9	10	0	8	0	8	–	*qnrB19*^(4)^	–	*qnrB19*^(4)^
	P10	10	0	9	0	9	–	–	–	–
	P11	10	0	10	0	10	–	–	–	–
	P12	10	0	7	0	7		*qnrB19*^(3)^	–	*qnrB19*^(3)^
	P13	10	8	9	0	19	${\mathrm{bla}}_{\text{CTX}-\text{M}-8}^{\left(3\right)}$, ${\mathrm{bla}}_{\text{CMY}-2}^{\left(3\right)}$, ${\mathrm{bla}}_{\text{SHV}-2\text{a}}^{\left(2\right)}$	*qnrB19*^(9)^, *qnrE1*^(2)^	–	${\mathrm{bla}}_{\text{CTX}-\text{M}-8}^{\left(3\right)}$, ${\mathrm{bla}}_{\text{SHV}-2\text{a}}^{\left(2\right)}$, *bla*_CMY−2_/*qnrB19*^(1)^, ${\mathrm{bla}}_{\text{CMY}-2}^{\left(2\right)}$ *qnrB19*^(7)^, *qnrB19*/*qnrE1*^(1)^, *qnrE1*^(1)^
	Total	132	23	105	1	140	23	62	1	
Swine	S1	10	6	8	0	17	${\mathrm{bla}}_{\text{CTX}-\text{M}-8}^{\left(6\right)}$	*qnrB19*^(4)^, *qnrS1*^(1)^	–	${\mathrm{bla}}_{\text{CTX}-\text{M}-8}^{\left(6\right)}$, *qnrB19*^(4)^, *qnrB19*/*qnrS1*^(1)^
	S2	10	7	8	0	17	${\mathrm{bla}}_{\text{CTX}-\text{M}-8}^{\left(1\right)}$, ${\mathrm{bla}}_{\text{CTX}-\text{M}-14}^{\left(3\right)}$, ${\mathrm{bla}}_{\text{CTX}-\text{M}-15}^{\left(1\right)}$, ${\mathrm{bla}}_{\text{CMY}-2}^{\left(2\right)}$	*qnrB19*^(8)^, *qnrS1*^(6)^	–	*bla*_CTX−M−15_/*qnrB19*/*qnrS1*^(1)^, ${\mathrm{bla}}_{\text{CTX}-\text{M}-14}^{\left(2\right)}$, ${\mathrm{bla}}_{\text{CMY}-2}^{\left(2\right)}$, *bla*_CTX−M−14_/*qnrB19*^(1)^, *qnrB19*/*qnrS*^(4)^, ${\mathrm{bla}}_{\text{CTX}-\text{M}-8}^{\left(1\right)}$, *qnrB19*^(2)^, *qnrS1*^(1)^
	S3	10	10	10	7	26	${\mathrm{bla}}_{\text{CMY}-2}^{\left(8\right)}$, ${\mathrm{bla}}_{\text{SHV}-12}^{\left(7\right)}$	*qnrB19*^(1)^	*mcr-1*^(7)^	${\mathrm{bla}}_{\text{SHV}-12}^{\left(7\right)}$, *mcr-1*^(5)^, *mcr-1*/${\mathrm{bla}}_{\text{CMY}-2}^{\left(2\right)}$, ${\mathrm{bla}}_{\text{CMY}-2}^{\left(6\right)}$, *qnrB19*^(1)^
	S4	10	5	10	0	17	${\mathrm{bla}}_{\text{CTX}-\text{M}-8}^{\left(4\right)}$, ${\mathrm{bla}}_{\text{CTX}-\text{M}-14}^{\left(1\right)}$	*qnrS1*^(8)^		${\mathrm{bla}}_{\text{CTX}-\text{M}-8}^{\left(4\right)}$, ${\mathrm{bla}}_{\text{CTX}-\text{M}-14}^{\left(1\right)}$, *qnrS1*^(8)^
	S5	10	8	10	0	22	${\mathrm{bla}}_{\text{CTX}-\text{M}-8}^{\left(7\right)}$	*qnrB19*^(3)^, *qnrS1*^(7)^		*bla*_CTX−M−8_/*qnrB19*^(1)^, *qnrB19*/*qnrS1*^(1)^, qnrS1^(6)^ *bla*_CTX−M−8_^(7)^
	Total	50	36	46	7	99	40	38	7	

*The number in parentheses corresponds to the number of isolates containing that gene or genotype. cro, Ceftriaxone; R, Resistance; cip, Ciprofloxacin; col, Colistin; PMQR, plasmid-mediated quinolone resistance*.

Pig and bovine feces were collected wearing latex gloves directly from animals; conversely, chicken samples were taken directly from cloacae with sterile swabs. All samples were refrigerated at 4°C and sent, within 24 h, to Departamento de Bacteriología y Virología (Instituto de Higiene, Montevideo, Uruguay), or to Plataforma de Investigación en Salud Animal for processing (Colonia, Uruguay).

Samples were pre-enriched in Luria Bertani broth for 12 h at 37°C. Next, 10 μl of the broth were cultured on MacConkey Agar plates (Oxoid Ltd., Basingstoke, UK) supplemented with 0.125 mg/L ciprofloxacin (ION, Montevideo, Uruguay) or 1 mg/L ceftriaxone (Libra, Montevideo, Uruguay), or 3 mg/L colistin (Sigma-Aldrich St. Louis MO USA).

### Identification and Antibiotic Susceptibility Testing

Putative *E. coli* colonies were identified by matrix assisted laser desorption ionization–time of flight (MALDI-TOF) mass spectrometry (Bruker, MA) in the facilities of Instituto de Higiene (Montevideo, Uruguay).

We then tested susceptibility to the following antibiotics: amoxicillin-clavulanic acid (AMC), ceftriaxone (CRO), ceftazidime (CAZ), ciprofloxacin (CIP), gentamicin (CN), amikacin (AK), and trimethoprim-sulfamethoxazole (SXT). Results were interpreted according to the Clinical Standard Laboratory Institute 2020 guidelines (15); isolates displaying intermediate resistance levels were considered as resistant.

Isolates showing resistance to 3rd generation cephalosporins underwent phenotypic screening for ESBLs and/or AmpC β-lactamases with the synergy double-disc method, using ESBL and AmpC inhibitors (AMC and boronic acid, respectively) plus 3rd generation cephalosporins according to Cordeiro et al. (16).

Minimum inhibitory concentration to colistin was determined for *E. coli* isolates recovered from MacConkey supplemented with the aforementioned antibiotic, by colistin agar test according to CLSI (15); *E. coli* ATCC 25922 was used as a quality control.

### Detection of ESBL, PMQR, and Colistin Resistance Genes

Resistance genes were sought by polymerase chain reaction (PCR) and confirmed by sequencing. We searched for the following resistance genes:

- oxyimino-cephalosporins
  *bla*_CTX−M−group−1_, *bla*_CTX−M−group−2_, *bla*_CTX−M−group−3_, *bla*_CTX−M−group−4_, *bla*_CTX−M−group−25_, *bla*_CTX−M−8_, *bla*_CTX−M−9_, *bla*_TEM_, *bla*_SHV_, *bla*_PER−2_, *bla*_AmpC_ (17);
- quinolones
  *aac (**15**) Ib-cr, qnrA, B, C, D, E, S, Vc, qepA* (17);
- colistin
  Detection of plasmid-mediated colistin resistance genes (mcr-1, mcr-2, and mcr-3) was performed by Real-Time PCR according to Li et al. (18) and mcr 4 was sought by PCR according to Carattoli et al. (19).
  The detailed list of primers used can be found in Supplementary Table 1.

## Results

### General Results

During the present work, 687 *E. coli* isolates were recovered: 334 from calves, 200 from poultry and 153 from pigs.

The 687 isolates were subjected to antibiotic susceptibility determination and detection of antibiotic resistance genes, as described in the materials and methods section. However, to avoid duplicate results, for each animal studied, only those isolates that presented phenotypic or genotypic differences are presented. Accordingly, we selected 73 bovine isolates, 141 poultry isolates and 99 pig isolates (Supplementary Table 2).

The most frequently detected resistance was to fluoroquinolones, being present in all of the analyzed farms. In 214/282 (75.9%) of the studied animals, we detected ciprofloxacin-resistant *E. coli* isolates (observed in 55 calves, 110 chickens, and 49 pigs) (Table 1). The farms displaying the highest levels of resistance corresponded to those of pig farming, yielding resistant isolates in 98% of the studied animals (49/50), followed by poultry farming with 83.3% (110/132), and lastly calf stables with 55% of the studied animals.

Conversely, resistance to oxyimino-cephalosporins was detected in 60/282 animals (21.4%), albeit quite heterogeneously: 72% of the sampled swine yielded resistant isolates (36/50) distributed along all the analyzed farms, followed by poultry farms where 17.4% of the sampled animals harbored resistant isolates (23/132), in 6/13 studied farms. Finally, a single calf harboring resistant isolates was detected among the studied establishments.

Resistance to colistin was the lowest of the three tested antibiotics, only 8 animals carried resistant isolates (2.8%); seven corresponded to pigs and the remaining case to a chicken.

### Detection of Resistance Genes

The most frequently detected resistance gene was *qnrB19*, which was present in *E. coli* isolates recovered from 87 animals belonging to the 3 production lines. Regarding PMQR genes, *qnrS1* was the second most frequent, being detected in 23 animals. Sparing a single case corresponding to a calf, the remaining 22 cases corresponded to isolates recovered from swine. The third gene in frequency was *qnrE1*, found in 8 isolates obtained from chickens and calves.

Regarding resistance to oxyimino-cephalosporins, 8 different β-lactamase genes were detected, the most frequent being *bla*_CTX−M−8_ and *bla*_CMY−2_ found in 22 and 19 animals, respectively; next, *bla*_CTX−M−2_ and *bla*_SHV−12_ were both detected in 7 animals, followed by *bla*_CTX−M−14_ in 4, *bla*_CTX−M−15_ and *bla*_SHV2a_ in 2, and *bla*_CTX−M−55_ in a single animal (Table 1).

Finally, transferable colistin resistance genes were detected in 2 establishments, belonging to a pig and a poultry farm; in the former, *mcr-1* was found in 8 animals, whereas in the latter in a single animal. In 3 pig-derived isolates, *mcr-1* was found along with *bla*_CMY−2_, while in the poultry isolate *mcr-1* was detected alongside *qnrB19*.

### Assessment of Relative Frequencies of Transferable Resistance Genes

As previously mentioned, in the present work we selected 73 *E. coli* isolates obtained from 100 calves, 141 isolates obtained from 132 chickens, and 99 isolates obtained from 50 pigs, thus yielding genotype/studied animal ratios of 0.73 (73/100), 1.08 (140/132), and 1.77 (99/50) for calves, poultry, and swine, respectively. Additionally, 30% of the *E. coli* resistant genotypes recovered from calves were linked to transferable resistance genes (22/73); conversely, in poultry, and swine, the frequency of isolates harboring transferable resistance genes was 55% (78/140) and 76% (75/99), respectively (Table 1).

## Discussion

This is the first work tackling the presence of transferable resistance to antibiotics considered as highest priority critically important antibiotics for human health in three chains of production animals in Uruguay.

The most alarming situation was observed in swine husbandry, where the highest percentage of animals harboring resistant isolates, the highest number of bacterial genotypes per animal and the highest percentage of transferable resistance genes were observed. Taking into account this scenario it is possible to hypothesize that, from the three populations studied, the swine digestive tract is where the best condition for resistance genes horizontal transfer events occur.

Recently, Van Boeckel et al. have reported that the global consumption of antibiotics per kilogram of animal in cattle, poultry, and swine husbandry is 45, 148, and 172 mg, respectively (7). Our data show some degree of correlation with that study, in the sense that a greater use of antibiotics entails a higher detection of resistance mechanisms on account of the selective pressure imposed.

Nevertheless, resistance to colistin (at least in this study) is lower than values reported in other countries of our region and the rest of the world, such as Ecuador, Argentina, and Spain (20–22). During this work we detected the presence of 8/50 pigs (16%) carrying *mcr-1-*harboring *E. coli*, all of them corresponding to the same farm. In 5/7 animals, the genes *mcr-1* and *bla*_CMY−2_ were found in separate *E. coli* isolates, yet in 3 animals those genes were found within the same isolate (Supplementary Table 2). Recently, we reported the detection of the first *E. coli* isolates of human origin carrying *mcr-1* in Uruguay. One of these isolates harbored *bla*_CMY−2_ and *mcr-1*, albeit in different plasmids; in this sense, *mcr-1* was encoded in an IncI2-type plasmid (23). More studies are needed to determine if there is a relationship between these isolates (i.e., *mcr-1*–*bla*_CMY−2_ bearing isolates of human and animal origin) or the genetic platforms that encode them. In addition, we also found an isolate obtained from poultry, carrying *mcr-1*. Presumably, the mandatory ban in Uruguay on veterinary usage of colistin, in any of its forms, will have beneficial effects by reducing the selection pressure on microorganisms carrying *mcr-1*. However, keeping in mind that *mcr* alleles are frequently associated with genes conferring resistance to other antibiotics widely used in veterinary medicine, such as quinolones and oxyimino cephalosporins, co-selection events are likely to occur. This has been demonstrated by the presence of the *mcr* gene along with ESBL, carbapenemases or plasmid-mediated quinolone resistance mechanisms such as *qnrB* or *qnrS* in different plasmids (IncI2, IncX4, y IncHI2) (24–27).

Regarding resistance to 3rd generation cephalosporins and quinolones in swine and poultry, the most frequently detected genes were *bla*_CTX−M−8_ and *bla*_CMY−2_, and *qnrB19*, respectively. A similar situation was found in human *Salmonella enterica* isolates in recent surveillance studies in our country (16). Since this microorganism is a primary pathogen and is associated with episodes of gastroenterocolitis, it could act as a doorway for resistance genes circulating in agricultural and veterinary environments. Notwithstanding, the intake of *E. coli* strains harboring the same or other resistance mechanisms could go unnoticed in the event of asymptomatic gut colonization.

In this concern, colonization with *E.coli* carrying resistance genes to antibiotics of critical use could be considered as a silent zoonosis, contributing to the resistance gene pool of microorganisms present in the gastrointestinal tract of animals and humans. It has been observed that gut colonization by *E. coli* strains with reduced susceptibility to fluoroquinolones can last from 2 weeks to 6 months, whereas the presence of β-lactamase-producing *E. coli* in outpatients can last up to 4 months in feces. Conversely, the persistence of ESBL-producing *E. coli* and *K. pneumoniae* in feces from recently discharged patients can last an average of 98 days (range 14–182 days) (28, 29).

Among the various ESBLs detected in poultry we also found *bla*_CTX−M−55_. This β-lactamase had never been reported in our country; yet in our neighboring country Brazil, it has been reported both in poultry and humans, usually associated to the glutathione transferase gene *fosA3*, responsible of conferring resistance to fosfomycin (30, 31). In order to assess this probable association in our poultry-derived isolates, we also performed PCR detection of *fosA3*, obtaining positive results (data not shown).

Although the main mechanism of resistance to oxyimino-cephalosporins in enterobacteria of human origin is *bla*_CTX−M−15_, this ESBL gene occasionally occurred in the isolates analyzed in the present work. In Uruguay *bla*_CTX−M−2_, *bla*_CTX−M−8_, *bla*_SHV−12_, and *bla*_SHV−2_ are among the most frequently detected β-lactamase genes, mainly in pediatric samples; more studies are needed to determine if there is any link with the microorganisms detected in this work or, perhaps, with the genetic structures that encode these genes (32–34).

In a previous report, we found *E. coli* isolates obtained from cattle showing some degree of resistance to fluoroquinolones, namely, 7.3% were carriers of PMQR (mainly *qnrB2* and *qnrS*) (14). In the present work, the number of isolates carrying PMQR genes rose to 31.5% (23/73); furthermore, we also detected a change in the circulating alleles. In this regard, gene *qnrB19* was the most frequently found, while the presence of *qnrE1* and *qnrS1* was detected in two and one isolate, respectively. The occurrence of *qnrE1* has been recently reported in isolates of *Salmonella enterica* from cattle in Brazil (35), however it has not been described outside the *Salmonella* genus in neither cattle nor poultry; thus, in this work we confirm the circulation of such gene in *E. coli* both in cattle and poultry.

The *qnrE1* gene was first reported in Argentina in 2017, in a human clinical isolate obtained in 2007 (36). Interestingly, in 2011 we reported the occurrence in Uruguay of a *qnrB* variant, termed *qnrBKp737* (defined by a partial sequence of 606 bp obtained from a PCR amplification product) (37). The *in silico* translation of the partial nucleotide sequence displayed 26 amino acid differences with QnrB1; further comparison showed that *qnrBKp737* was identical to *qnrE1* (unpublished data). Apparently, this resistance mechanism scarcely reported so far, is also a long-standing problem in the context of “One Health” in our region.

One limitation of our work is the fact that we did not conduct a study of risk factors to determine which variables influence the selection of resistant microorganisms.

In light of our results, it will be necessary to carry out new studies encompassing a greater number of establishments with a design that allows us to analyze these aspects.

Beyond these limitations, the wide distribution of fluoroquinolone-resistant isolates in the animals analyzed is alarming and may reflect the widespread use of antimicrobials such as enrofloxacin. Since this class of antibiotics is on the list of highest priority critically important antibiotics for human health, their use in veterinary medicine should be drastically limited.

In conclusion, we have detected transferable resistance genes to the three antibiotics considered critical to human health, present in feces from farm animals in Uruguay. Several of such genes have also been reported previously in microorganisms of human origin in our country. Tackling the problem of antimicrobial resistance requires comprehensive approaches, including prudent use of antibiotics and surveillance under the “One Health” concept.

## Data Availability Statement

The raw data supporting the conclusions of this article will be made available by the authors, without undue reservation.

## Author Contributions

NC, AU, and PÁ: carrying out of antibiotic sensitivity studies and detection of resistance genes by PCR and preparation of the manuscript. BF: pig samples collection, antibiotic sensitivity studies, and detection of resistance genes by PCR. NFC: real-time PCR for mcr-1, 2, and 3, interpretation of results, and preparation of the manuscript. GT: poultry samples collection, interpretation of results, and preparation of the manuscript. GC: pig samples collection, interpretation of results, and preparation of the manuscript. MC and MF: calves sample collection, interpretation of results, and preparation of the manuscript. PZ: experimental design, general coordination, interpretation of results, and elaboration of the manuscript. IB and RV: experimental design, general coordination, sequence analysis, interpretation of results, and elaboration of the manuscript. All authors contributed to the article and approved the submitted version.

## Conflict of Interest

The authors declare that the research was conducted in the absence of any commercial or financial relationships that could be construed as a potential conflict of interest.
