# Supplementary material for: Transferable Resistance to Highest Priority Critically Important Antibiotics for Human Health in Escherichia coli Strains Obtained From Livestock Feces in Uruguay
Source: Front Vet Sci. 2020 Nov 19;7:588919. doi: 10.3389/fvets.2020.588919 (PMC7717973; doi:10.3389/fvets.2020.588919)
Supplement: Supplementary file 1 [file Table_1.DOCX]

**table S1. List of primers used in this work:**

| **Colistin** | | | | | | |
| --- | --- | --- | --- | --- | --- | --- |
| **Gene** | **Primer name** | **Sequence 5’-3’** | **Product size** | | **Annealing Temp.** | **Control** |
| ***mcr-1*** | **mcr1-qf** | **AAAGACGCGGTACAAGCAAC** | **213 bp** | | **60 ºC** | **Li *et al.,* 2017** |
|  | **mcr1-qr** | **GCTGAACATACACGGCACAG** |  |  |  |  |
| ***mcr-2*** | **mcr2-qf** | **CGACCAAGCCGAGTCTAAGG** | **92 bp** | |  |  |
|  | **mcr2-qr** | **CAACTGCGACCAACACACTT** |  |  |  |  |
| ***mcr-3*** | **mcr-3 qf** | **ACCTCCAGCGTGAGATTGTTCCA** | **169 bp** | |  |  |
|  | **mcr-3 qr** | **GCGGTTTCACCAACGACCAGAA** |  |  |  |  |
| **Cycle of 50°C for 2min, 95°C for 3min, then 40 cycles of 95°C for 30s, 60°C for 30s, and 72°C for 30s, followed by a 72°C to 95°C ramp for the melting curve resolution.** | | | | | | |
| ***mcr-4*** | **mcr-4 F** | **ATTGGGATAGTCGCCTTTTT** | **487 bp** | **52 ºC** | | **Carattoli *et al*., 2017** |
|  | **mcr-4 R** | **TTACAGCCAGAATCATTATCA** |  |  |  |  |
| **Program: 1) 94º-5min. 2) 94º - 40s / annealing temp. - 40s / 72º - 40s x 35 cycles              3) 72º - 10min.** | | | | | | |
| **AmpC** | | | | | | |
| **Gene** | **Primer name** | **Sequence 5’-3’** | **Product size** | | **Annealing Temp.** | **Control** |
| **CIT (CMY)** | **CIT F** | **TGGCCAGAACTGACAGGCAAA** | **461 bp** | | **64°C** | **García Fulgueiras *et al*., 2019; Cordeiro *et al*., 2016; Perez-Perez & Hanson, 2002.** |
|  | **CIT R** | **TTTCTCCTGAACGTGGCTGGC** |  |  |  |  |
| **DHA** | **DHA F** | **AACTTTCACAGGTGTGCTGGGT** | **404 bp** | | **64°C** |  |
|  | **DHA R** | **CCGTACGCATACTGGCTTTGC** |  |  |  |  |
| **MOX** | **MOX F** | **GCTGCTCAAGGAGCACAGGAT** | **519 bp** | | **64°C** |  |
|  | **MOX R** | **CACATTGACATAGGTGTGGTGC** |  |  |  |  |
| **ACC** | **ACC F** | **AACAGCCTCAGCAGCCGGTTA** | **345 bp** | | **64°C** |  |
|  | **ACC R** | **TTCGCCGCAATCATCCCTAGC** |  |  |  |  |
| **EBC** | **EBC F** | **TCGGTAAAGCCGATGTTGCGG** | **309 bp** | | **64°C** |  |
|  | **EBC R** | **CTTCCACTGCGGCTGCCAGTT** |  |  |  |  |
| **FOX** | **FOX F** | **AACATGGGGTATCAGGGAGAT** | **189 bp** | | **64°C** |  |
|  | **FOX R** | **CAAAGCGCGTAACCGGATTGG** |  |  |  |  |
| **Program: 1) 94º-3:30 min.         2) 94º - 30s / annealing temp. - 30s /72º - 1min. x 25 cycles                 3) 72º - 7min.** | | | | | | |
| \| **ESBLs** \| \| \| \| \| \| \| --- \| --- \| --- \| --- \| --- \| --- \| \| **Gene** \| **Primer name** \| **Sequence 5-3’** \| **Product size** \| **Annealing Temp.** \| **Control** \| \| ***bla*_SHV_** \| **SHV A** \| **ATGATGAGCACCTTTAAAGTA** \| **620 bp** \| **52ºC** \| **RAM VICky** \| \| **SHV B** \| **ATTTCGCTCGGCCATGCTCGC** \| \| ***bla*_PER_-2** \| **PER 2 F** \| **TGTGTTTTCACCGCTTCTGCTCTG** \| **878 bp** \| **48ºC** \| **Vignoli R *et al*., 2005** \| \| **PER 2 R** \| **CAGCTCAAACTGATAAGCCGCTTG** \| \| ***bla*_TEM_** \| **OT3** \| **ATGAGTATTCAAACTTTCCG** \| **900 bp** \| **52ºC** \| **Vignoli R *et al*., 2006** \| \| **OT4** \| **CCACTGCTTAATCAGTGAGG** \| \| **PAN-CTX-M** \| **PANCTX M F** \| **TTTGCGATGTGCAGTACCAGTAA** \| **543 bp** \| **59ºC** \| **Moyá *et al*., 2012** \| \| **PAN CTX M R** \| **CGATATCGTTGGTGGTGCCATA** \| \| ***bla*_CTX-M_ (gr1)** \| **CTX-M-1 F** \| **CCCATGGTTAAAAAATCACTG** \| **830 bp (F-AS)**  **450 bp(F-R)** \| **54ºC** \| **RAM VICky** \| \| **CTX-M-1 R** \| **GCTAGCCGGGCCGCCAACGTGA** \| \| **CTX-M- AS R** \| **CGTAGCCGGGCCGCCAACGTGA** \| \| ***bla*_CTX-M_ (gr2)** \| **CTX-M gr2 F** \| **TTAATGATGACTCAGAGCATT** \| **875 bp** \| **51ºC** \| **RAM VICky**    **RAM VICky**  **RAM VIcky**  **Marcano et al. 2011**  **McGettigan et al. 2009** \| \| **CTX-M gr2 R** \| **GATACCTCGCTCCATTTATTGC** \| \| ***bla*_CTX-M_ (gr3)** \| **CTX-M gr3 F** \| **ACCTGATTAACTACAATCCCAT** \| **514 bp** \| **55ºC** \| \| **CTX-M gr3 R** \| **ACTTTCTGCCTTCTGCTCTGGC** \| \| ***bla*_CTX-M_ (gr4)** \| **CTX-M gr4 F** \| **GCTGGAGAAAAGCAGCGGAG** \| **473 bp** \| **57ºC** \| \| **CTX-M gr4 R** \| **GTAAGCTGACGCAACGTCTG** \| \| ***bla*_CTX-M-8_ (gr3)** \| **CTX-M-8 F** \| **TGAATACTTCAGCCACACG** \| **922 bp** \| **50ºC** \| \| **CTX-M-8 R** \| **TAGAATTAATAACCGTCGGT** \| \| ***bla*_CTX-9-M_ (gr4)** \| **CTX-9-M F** \| **ATGGTGACAAAGAGARTGCAA** \| **841 bp** \| **60ºC** \| \| **CTX-9-M R** \| **TTACAGCCCTTCGGCGATGAT** \| \| ***bla*_CTX-M_ (gr25)** \| **CTX-M-25 F** \| **AGAAAAAGCGTAAGGCGGGC** \| **862 bp** \| **56ºC** \| **RAM VICky** \| \| **CTX-M-25 R** \| **CCGTCGGTGACAATTCTGGC** \| \| **CTX-M groups: gr1 - 1, 3, 10, 12, 15/ gr2 - 2, 4, 5, 6, 7, 20/ gr3 – 8/ gr4 - 9, 13, 14, 16, 17, 19, 21, 27/ gr25 - 25, 26** \| \| \| \| \| \| \| **Program: 1) 94º-5min.         2) 94º - 1min. / annealing temp. - 1min. /72º - 1min. x 30 cycles                  3) 72º - 5min.** \| \| \| \| \| \| \| \| **Quinolone** \| \| \| \| \| \| \| --- \| --- \| --- \| --- \| --- \| --- \| \| **Gene** \| **Primer name** \| **Sequence 5’-3’** \| **Product size** \| **Annealing Temp.** \| **Control** \| \| ***qnrA*** \| **qnrA f** \| **ATTTCTCACGCCAGGATTTG** \| **516 bp** \| **53 ºC** \| **RAM Vicky**  **RAM VICky**  **RAM VICky**  **RAM VICky**  **RAM VICky**  **RAM VICky**  **RAM VICky** \| \| **qnrA r** \| **GATCGGCAAAGGTTAGGTCA** \| \| ***qnrB*** \| **qnrB f** \| **GATCGTGAAAGCCAGAAAGG** \| **469 bp** \| **52 ºC** \| \| **qnrB r** \| **ACGACGCCTGGTAGTTGTCC** \| \| ***qnrC*** \| **qnrC f** \| **GGGTTGTACATTTATTGAATC** \| **307 bp** \| **50 ºC** \| \| **qnrC r** \| **CACCTACCCATTTATTTTCA** \| \| ***qnrD*** \| **qnrD f** \| **CGAGATCAATTTACGGGGAATA** \| **580 bp** \| **51 ºC** \| \| **qnrD r** \| **AACAAGCTGAAGCGCCTG** \| \| ***qnrS*** \| **qnrS f** \| **ACGACATTCGTCAACCTGCAA** \| **417 bp** \| **53 ºC** \| \| **qnrS r** \| **TAAATTGGCACCCTGTAGGC** \| \| ***qnrE*** \| **qnrE1-F** \| **GGCATTGATTTTTGAAGGCGA** \| **516 bp** \| **55 ºC** \| \| **qnrE1-R** \| **GTGGGTAAAATTGGCCGCTC** \| \| ***qnrVC*** \| **qnrVC f** \| **ATGGAAAAATCAAAGCAATT** \| **656 bp** \| **46ºC** \| \| **qnrVC r** \| **TTAGTCAGGAACAATGATTA** \| \| ***qepA*** \| **qepA f** \| **AACTGCTTGAGCCCGTAGAT** \| **595 bp** \| **56 ºC** \| \| **qepA r** \| **GTCTACGCCATGGACCTCAC** \| \| ***aac(6’)-Ib-cr*** \| **aacF**  **aacR** \| **TTGCGATGCTCTATGAGTGGCTA**  **CTCGAATGCCTGGCGTGTTT** \| **481 bp** \| **55°C** \| **RAM VICky** \| \| **Program: 1) 94º-5min.        2) 94º - 1min. / annealing temp. - 1min. / 72º - 1min. x 30 cycles                  3) 72º - 7min.** \| \| \| \| \| \| \| **Fosfomycin** \| \| \| \| \| \| \| **Gene** \| **Primer name** \| **Sequence 5’-3’** \| **Product size** \| **Annealing Temp.** \| **Control** \| \| ***fosA3*** \| **fosA3f**  **fosA3R** \| **TGAATCATCTGACGCTGG**  **TCAATCAAAAAAGACCATC** \| **404 bp** \| **55°C** \| **Ram Vicky** \| \| **Program: 1) 98º-3min.        2) 98º - 10s / annealing temp. - 30s / 72º - 1min. x 30 cycles                  3) 72º - 5min.** \| \| \| \| \| \| \| \| \| \| \| \| | | | | | | |

**References**

- Carattoli A, Villa L, Feudi C, Curcio L, Orsini S, Luppi A, et al. 2017. Novel plasmid-mediated colistin resistance *mcr-4* gene in Salmonella and *Escherichia coli*, Italy 2013, Spain and Belgium, 2015 to 2016. Eurosurveillance. 3;22(31):30589
- Chen, X., Zhang, W., Pan, W., Yin, J., Pan, Z., Gao, S., & Jiao, X. 2012. Prevalence of qnr, aac(6′)-Ib-cr, qepA, and oqxAB in Escherichia coli isola...: Discovery Service para Timbó. *Antimicrobial Agents and Chemotherapy, 2012 June*, *56*(6), 3423–3427. <http://doi.org/0.1128/AAC.06191-11>
- Cordeiro NF, Nabón A, García-Fulgueiras V, Álvez M, Sirok A, Camou T, et al. 2016. Analysis of plasmid mediated quinolone and oxyimino-cephalosporin resistance mechanisms in Uruguayan Salmonella enterica isolates from 2011 to 2013. J Glob Antimicrob Resist 6:165–171.
- Garcia-Fulgueiras V, Zapata Y, Papa-Ezdra R, Ávila P, Caiata L, Seija V, et al. 2019. First characterization of *K. pneumoniae* ST11 clinical isolates harboring *blaKPC-3* in Latin America. Rev Argent Microbiol. 21:S0325-7541(19)30113-0
- Garcia-Fulgueiras V, Araujo L, Bado I, Cordeiro NF, Mota MI, Laguna G, et al. 2017. Allodemic distribution of plasmids co harbouring CTX-M-15/Aac(6’)Ib-cr/QnrB in *K. pneumoniae* is the main source of ESBL in Uruguay’s Paediatric Hospital. J Glob Antimicrob Resist 9:68-73.
- Li J, Shi X, Yin W, Wang Y, Shen Z, Ding S, et al. 2017. A multiplex SYBR green real-time PCR assay for the detection of three colistin resistance genes from cultured bacteria, feces, and environment samples. Front Microbiol 27;8:2078.
- Moyá, B., Beceiro, A., Cabot, G., Juan, C., Zamorano, L., Alberti, S., & Oliver, A. 2012. Pan-β-lactam resistance development in Pseudomonas aeruginosa clinical strains: molecular mechanisms, penicillin-binding protein profiles, and binding affinities. *Antimicrobial Agents and Chemotherapy*, *56*(9), 4771–4778.<https://doi.org/10.1128/AAC.00680-12>
- Perez-Perez, F. J., & Hanson, N. D. 2002. Detection of Plasmid-Mediated AmpC -Lactamase Genes in Clinical Isolates by Using Multiplex PCR. *Journal of Clinical Microbiology*, *40*(6), 2153–2162.<https://doi.org/10.1128/JCM.40.6.2153-2162.2002>
- Vignoli, R., Varela, G., Mota, M. I., Cordeiro, N. F., Power, P., Ingold, E., Gadea, P., Sirok, A., Schelotto, F., Ayala, J. A., & Gutkind, G. 2005. Enteropathogenic Escherichia coli Strains Carrying Genes Encoding the PER-2 and TEM-116 Extended-Spectrum -Lactamases Isolated from Children with Diarrhea in Uruguay. *Journal of Clinical Microbiology*, *43*(6), 2940–2943.<https://doi.org/10.1128/JCM.43.6.2940-2943.2005>
- Vignoli, Rafael, Cordeiro, N. F., García, V., Mota, M. I., Betancor, L., Power, P., Chabalgoity, J. A., Schelotto, F., Gutkind, G., & Ayala, J. A. 2006. New TEM-Derived Extended-Spectrum-Lactamase and Its Genomic Context in Plasmids from Salmonella enterica Serovar Derby Isolates from Uruguay. *ANTIMICROBIAL AGENTS AND CHEMOTHERAPY*, *50*(2), 781–784.<https://doi.org/10.1128/AAC.50.2.781-784.2006>
- Yang YQ, Li YX, Lei CW, Zhang AY, Wang HN. 2018. Novel plasmid-mediated colistin resistance gene *mcr-7.1* in *Klebsiella pneumoniae*. J Antimicrob Chemother. 1;73(7):1791-1795.
- Yossapol, M., Sugiyama, M., & Asai, T. 2017. The occurrence of CTX-M-25-producing Enterobacteriaceae in day-old broiler chicks in Japan. *J. Vet. Med. Sci*, *79*(10), 1644–1647.<https://doi.org/10.1292/jvms.17-0294>
- Prevalence of CTX-M β-Lactamases in Philadelphia, Pennsylvania. Shannon E. McGettigan, Baofeng Hu, Kathleen Andreacchio, Irving Nachamkin, Paul H. Edelstein. Journal of Clinical Microbiology Aug 2009, 47 (9) 2970-2974; DOI: 10.1128/JCM.00319-09.
- Marcano, Daniel,De Jesús, Andreína,Hernández, Luis,Torres, Luis (2011) Frecuencia de enzimas asociadas a sensibilidad disminuida a betalactámicos en aislados de enterobacterias, Caracas, Venezuela. Rev Panam Salud Publica;30(6),dec. 2011.
